# Activity-independent targeting of mTOR to lysosomes in primary osteoclasts

**DOI:** 10.1038/s41598-017-03494-2

**Published:** 2017-06-07

**Authors:** Andrew Wang, Luciene R. Carraro-Lacroix, Celeste Owen, Bowen Gao, Paul N. Corey, Pascal Tyrrell, John H. Brumell, Irina Voronov

**Affiliations:** 1 Faculty of Dentistry, University of Toronto, Toronto, ON Canada; 2 Centre for Modeling Human Disease, Samuel Lunenfeld-Tanenbaum Research Institute, Mount Sinai Hospital, Toronto, ON Canada; 3 Department of Statistical Sciences, University of Toronto, Toronto, ON Canada; 4 Department of Medical Imaging, University of Toronto, Toronto, ON Canada; 5 Cell Biology Program, The Hospital for Sick Children, Toronto, ON Canada; 6 Department of Molecular Genetics, University of Toronto, Toronto, ON Canada; 7 Institute of Medical Science, University of Toronto, Toronto, ON Canada

## Abstract

Mammalian target of rapamycin (mTOR) is activated by numerous stimuli, including amino acids and growth factors. This kinase is part of the mTOR complex 1 (mTORC1) which regulates cell proliferation, differentiation, and autophagy. Active mTORC1 is located on lysosomes and has been reported to disassociate from the lysosomal surface in the absence of amino acids. Furthermore, mTORC1 activity has been linked to the vacuolar H^+^-ATPases (V-ATPases), the proton pumps responsible for lysosomal acidification; however, the exact role of the V-ATPases in mTORC1 signaling is not known. To elucidate the mechanisms involved in mTORC1 regulation by the V-ATPases, we used primary osteoclasts derived from mice carrying a point (R740S) mutation in the *a*3 subunit of the V-ATPase. In these cells, the mutant protein is expressed but the pump is not functional, resulting in higher lysosomal pH. By analyzing mTOR activation, mTOR/lysosome co-localization, and lysosomal positioning using confocal microscopy, fractionation, and ultrapure lysosomal purification methods, we demonstrate that in primary osteoclasts, mTOR is localized on the lysosomal surface even when mTOR activity is inhibited. Our findings reveal that mTOR targeting to the lysosome in osteoclasts is activity-independent, and that its disassociation from the lysosome during starvation is not universal.

## Introduction

Mammalian target of rapamycin (mTOR) is a serine/threonine kinase responsible for cellular responses to growth factors and nutrient availability. In cells, it exists as part of two complexes, complex 1 (mTORC1) and complex 2 (mTORC2)^1, 2^. mTORC1 activity is tightly linked to autophagy, a lysosomal degradation process necessary for maintenance of cellular homeostasis by ‘recycling’ of cellular proteins and organelles. In the absence of amino acids, inactive mTORC1 allows initiation of autophagic protein degradation to upregulate intracellular free amino acid levels. In the presence of amino acids, active mTORC1 suppresses autophagy and promotes cell proliferation, differentiation, and growth^3^.

mTORC1 regulation is tightly controlled and closely connected to the lysosomes and to the vacuolar H^+^-ATPases (V-ATPases)^4^. It has been shown that in the presence of amino acids, active mTOR translocates to the lysosome, while in the absence of amino acids, inactive mTOR disassociates from the lysosome (becomes cytosolic)^4^. Lysosomal positioning (peripheral *vs*. perinuclear) has also been reported to play a role in mTORC1 regulation: peripheral positioning correlates with active mTORC1, while amino acid deprivation and mTORC1 inactivation leads to perinuclear positioning^5^. In addition, the V-ATPases, the proton pumps responsible for maintenance of lysosomal pH, are also involved in mTORC1 regulation: inhibition of the V-ATPases by siRNA or by specific inhibitors decreases mTORC1 activity, confirming the importance of the V-ATPases in mTORC1 signaling. Furthermore, it has been shown in HEK-293T cells that active mTORC1 attaches to the lysosome *via* the Ragulator complex, and these Ragulator complex proteins, in turn, directly interact with several subunits of the V-ATPase^4, 6^. It has been proposed that the V-ATPases serve as amino acid sensors, controlling mTORC1 activation *via* the ‘unknown inside-out mechanism’^3^; however, the precise role of the V-ATPases in mTORC1 signaling has not been elucidated.

The V-ATPases are ubiquitously expressed multi-subunit proton pumps involved in maintenance of intracellular/organellar and extracellular pH^7^. These pumps consist of at least 14 different subunits which are divided into two distinct domains: the ATP-hydrolyzing V_1_ domain (subunits A, B, C, D, E, F, G, H), and the proton-translocating V_0_ domain (subunits *a*, *c*, *c′*, *c″*, *d* and *e*). Some of the V-ATPase subunits have several isoforms and these isoforms are cell-type and tissue specific. For example, the V_0_ domain ‘*a*’ subunit, the subunit responsible for proton translocation, has four isoforms, *a*1, *a*2, *a*3 and *a*4. The *a*1 isoform is highly expressed in neurons, while the *a*2 subunit is endosomal and is considered to be ubiquitous; the *a*4 isoform is found primarily in renal intercalated cells and epididymal clear cells. The V-ATPases containing the *a*3 subunit, although also ubiquitously expressed, are enriched over 100 fold in osteoclasts^7, 8^, the bone resorbing cells.

Osteoclasts are terminally differentiated multinucleated cells formed by fusion of hematopoietic precursors^9^. To resorb bone, osteoclasts change their morphology and form a unique ‘ruffled border’ membrane, a convoluted membrane structure adjacent to the bone surface. This ‘ruffled border’ membrane is enriched with the *a*3-containing V-ATPases, where these proton pumps acidify the area of resorption to dissolve the mineral component of bone and to create optimal environment for the enzymes to digest bone matrix proteins^9^. In non-resorbing osteoclasts, the *a*3-containing V-ATPases are located in the lysosomes and are responsible for maintaining lysosomal pH in these cells^10^. Mutations in the *a*3 subunit cause autosomal recessive osteopetrosis, a rare genetic disease characterized by dense but brittle bones due to inability of the osteoclasts to resorb bone^11^. There are several mouse models of autosomal recessive osteopetrosis that involve mutations or deletions of *a*3^12–14^; however, only the osteoclasts derived from mice with a point mutation in the *a*3 subunit of the V-ATPase have been reported to have an altered lysosomal pH^15^. In this mouse model, an evolutionary conserved arginine responsible for proton translocation across the membrane in *a*3 is mutated to serine (R740S), making the pump inactive: the mutant *a*3 protein is expressed and is targeted to the lysosomal membrane; however, its activity is significantly impaired^14, 15^. Lysosomal pH in heterozygous (+/R740S) and homozygous (R740S/R740S) osteoclasts is higher compared to the wild type (+/+) cells^15, 16^. This gene-dosage dependent ‘pre-set’ lysosomal pH in osteoclasts (4.5 vs. 5.7 vs. 6.2, in +/+, +/R740S, and R740S/R740S cells, respectively^16^) creates a unique *in vitro* model to study the role of the V-ATPases and lysosomal pH in signaling regulation in primary cells.

We have previously demonstrated that mTORC1 activity was significantly upregulated in +/R740S osteoclasts compared to the +/+ cells^17^, suggesting that lysosomal pH also plays a role in mTORC1 regulation. We decided to utilize the unique property of this ‘naturally’ pre-set high lysosomal pH of the R740S osteoclast model to decipher the role of lysosomal pH and the V-ATPases in mTORC1 regulation and lysosomal positioning. Here, we show that in primary osteoclasts, lysosomal pH does play a role in mTORC1 activation; however, the amino acid status does not affect mTOR co-localization with the lysosome, suggesting that mTORC1 regulation is not universal and is different in highly specialized cells, such as osteoclasts.

## Results

### mTORC1 activity is increased in R740S/R740S osteoclasts

**Figure 1:**
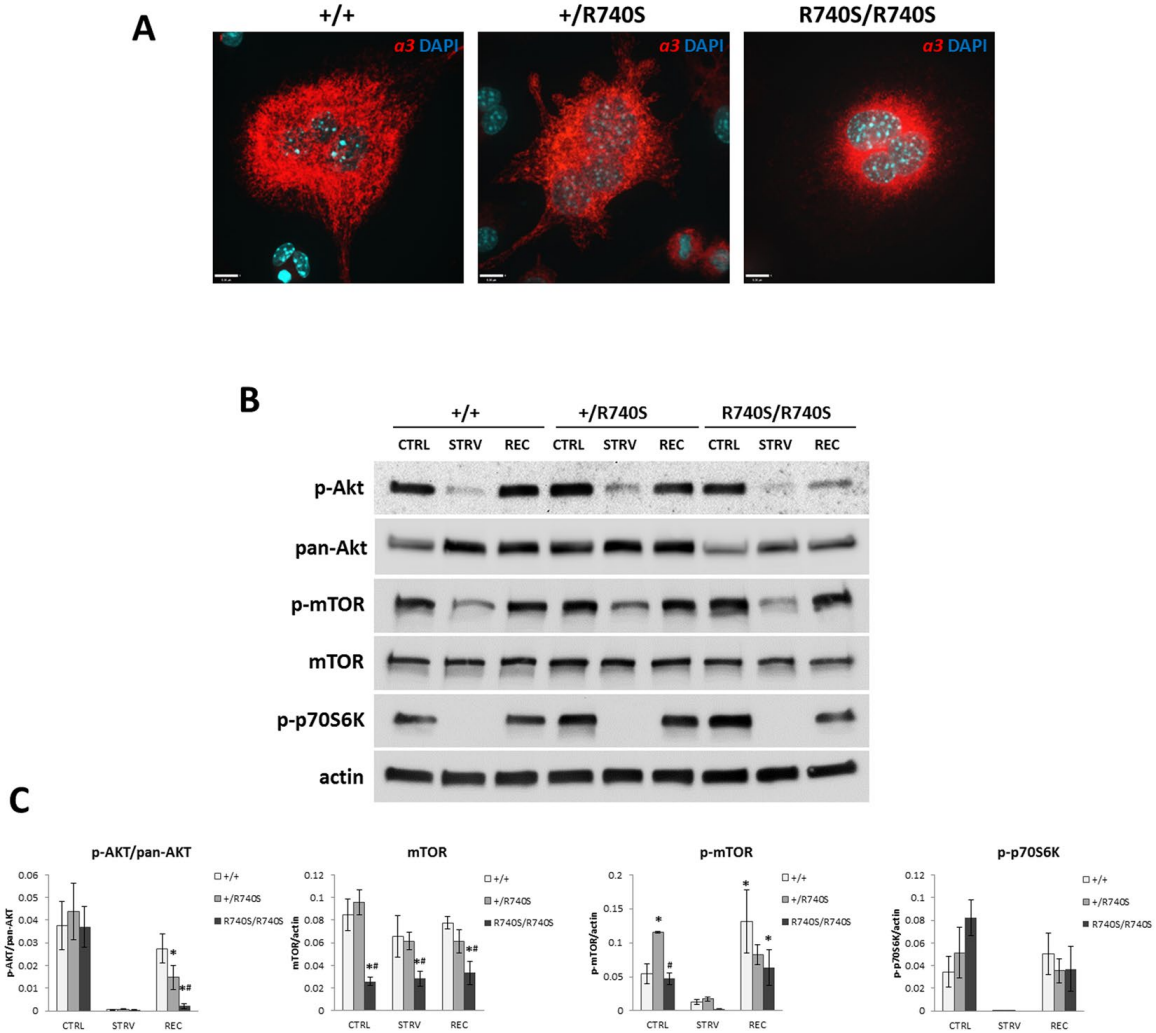
mTORC1 activity is increased in R740S/R740S cells. Spleen-derived osteoclasts were differentiated as described in “Materials and Methods”. On day 4 of culture, the cells were incubated with HBSS (starvation) for 60 min, and then in fully supplemented media for additional 30 min. (**A**) Immunofluorescence. Cells were fixed and stained with an anti-*a*3 antibody as described in “Materials and Methods”; nuclei were stained with DAPI. Representative images of three independent experiments. Bars, 15 μm. (**B**) Immunoblotting, representative cropped blots of 4 independent experiments. Whole cell lysates were separated on 4–20% gradient gels, transferred to a nitrocellulose membrane and probed for mTOR, p-mTOR, p-AKT, pan-AKT, p-p70S6K, and actin. (C) Quantification of immunoblots; combined normalized data of at least 4 independent experiments, mean ± std. dev; * indicates statistical significance compared to +/+ control, # indicates statistical significance compared to +/R740S control, p < 0.05.

We have previously shown that the V-ATPases containing the R740S mutation in the *a*3 subunit are not functional^14–16^. The mutant *a*3 protein R740S is expressed (Fig. 1A) and is localized to the lysosomes^15^. Using bone-marrow derived +/R740S osteoclasts, we have demonstrated that mTORC1 activity is increased^17^. To elucidate the connection between lysosomal pH, V-ATPases, and mTORC1 activity, we examined mTORC1 pathway in homozygous R740S/R740S osteoclasts. Due to severe osteopetrosis and an early lethality in these animals, osteoclasts were derived from the spleens of the 5–6 day old mice. We assessed mTORC1 activation and function by measuring the levels of total mTOR, p-AKT, p-mTOR, and phosphorylation of p70S6 kinase (p-p70S6K) under basal, starvation (HBSS, 1 hr), and recovery conditions. Assessing p-AKT levels represents signaling upstream of mTORC1, while p-mTOR represents signaling downstream of AKT, and p-p70S6K, in turn, is a well-characterized mTORC1 substrate.

Quantification of the immunoblots demonstrated that the R740S/R740S osteoclasts had significantly lower mTOR protein levels compared to the other two genotypes in all three conditions (Fig. 1B,C). We also observed that the basal levels of p-mTOR were increased in +/R740S spleen osteoclasts, confirming our previous results^17^, but not in R740S/R740S cells. The response to ‘starvation’ condition was identical in all three genotypes; however, the V-ATPase *a*3 mutation did have an effect on mTORC1 signaling in +/R740S and R740S/R740S cells (Fig. 1B,C). The basal levels of p-AKT (relative to pan-AKT) in +/R740S and R740S/R740S cells were similar to +/+; however, upon ‘recovery’, the levels of p-AKT in both genotypes did not recover to the basal levels and were lower compared to the +/+ cells. The basal levels of p-p70S6K, although they did not reach statistical significance (p = 0.056), were trending towards higher mTORC1 activity in the R740S/R740S cells. Taking into account the lower mTOR protein levels in R740S/R740S osteoclasts, the levels of p-p70S6K suggested a more active basal mTORC1 activity in R740S/R740S cells compared to the +/+ osteoclasts. Since, to our knowledge, lysosomal pH was the only factor altered in these cells, our results confirmed that the lysosomal pH plays a role in regulation of mTORC1 activity, affecting phosphorylation of both upstream (AKT) and downstream (p-mTOR, p-p70S6K) molecules involved in mTORC1 signaling.

### Autophagy is inhibited in osteoclasts with R740S mutation

**Figure 2:**
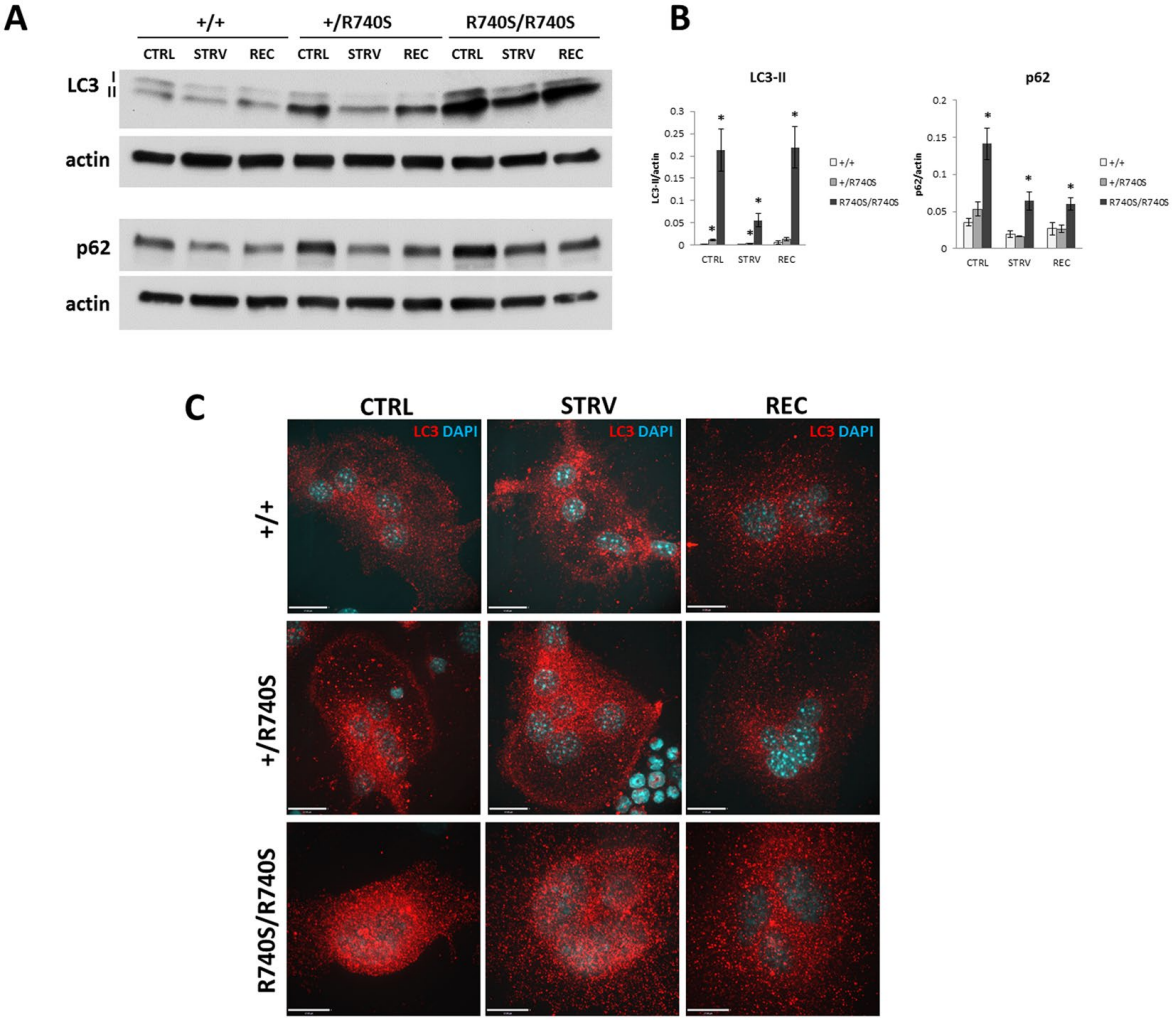
Autophagy is inhibited in osteoclasts with R740S mutation. Spleen-derived osteoclasts were differentiated as described in “Materials and Methods”. (**A**) Immunoblotting, representative cropped blots of 4 independent experiments. On day 4 of culture, the cells were incubated with HBSS (starvation) for 60 min, and then in fully supplemented media for additional 30 min. Whole cell lysates were separated on 4–20% gradient gels, transferred to a nitrocellulose membrane and probed for LC3, p62 and actin. (**B**) Quantification of immunoblots; combined normalized data of 4 independent experiments, mean ± std. dev; * indicates statistical significance compared to +/+ control, p < 0.05. (**C**) LC3 immunostaining. Spleen-derived osteoclasts were treated as described in (**A**), fixed, stained with anti-LC3B antibody as described in “Materials and Methods”; nuclei were stained with DAPI. Representative images. Bars, 17 μm.

To confirm that the R740S mutation affects lysosomal function in R740S/R740S osteoclasts, we assessed autophagy, a cellular pathway dependent on lysosomal degradation. We measured the levels of autophagy markers microtubule-associated protein light chain 3 (LC3) and p62 (also known as sequestosome 1/SQSTM1) in these cells. The lipidated form of LC3 (LC3-II) is a component of the autophagosomal membrane, while p62 is a chaperone responsible for delivering autophagic cargo to the autophagosomes for degradation. As autophagy proceeds, the levels of LC3-II and p62 decrease with time, making these two proteins convenient markers to assess the status of autophagic degradation^18^. To assess autophagy, the cells were starved for 1 hr (HBSS) and then recovered in fully supplemented media for 30 min. Both +/R740S and R740S/R740S cells had significantly higher protein levels of LC3-II compared to the +/+ controls (Fig. 2A,B), suggesting that the autophagy in these cells was inhibited as a result of higher lysosomal pH. Quantification of the immunoblots showed that the levels of p62 were significantly increased in R740S/R740S but not in +/R740S cells compared to +/+ controls. During starvation, the protein levels of both LC3-II and p62 had decreased in +/R740S and R740S/R740S cells, indicating that a certain amount of autophagic degradation still occurred and the autophagy was not completely blocked in R740S/R740S osteoclasts. Immunofluorescence staining of the osteoclasts with an anti-LC3B antibody had higher levels of autophagosome formation in the R740S/R740S cells (Fig. 2C). To confirm that LC3-II and p62 accumulation in +/R740S and R740S/R740S cells was due to higher lysosomal pH, the cells were incubated with chloroquine for 2 hrs. As expected, chloroquine treatment increased LC3-II and p62 levels in +/+ cells, but failed to do so in R740S/R740S cells (Figure S1). Collectively, our results confirmed that the *a*3 R740S mutation inhibits V-ATPase function in R740S/R740S, and partially in +/R740S osteoclasts, leading to decreased/blocked autophagic degradation in these cells.

### mTOR co-localization with the lysosome in osteoclasts does not change in response to amino acid availability

**Figure 3:**
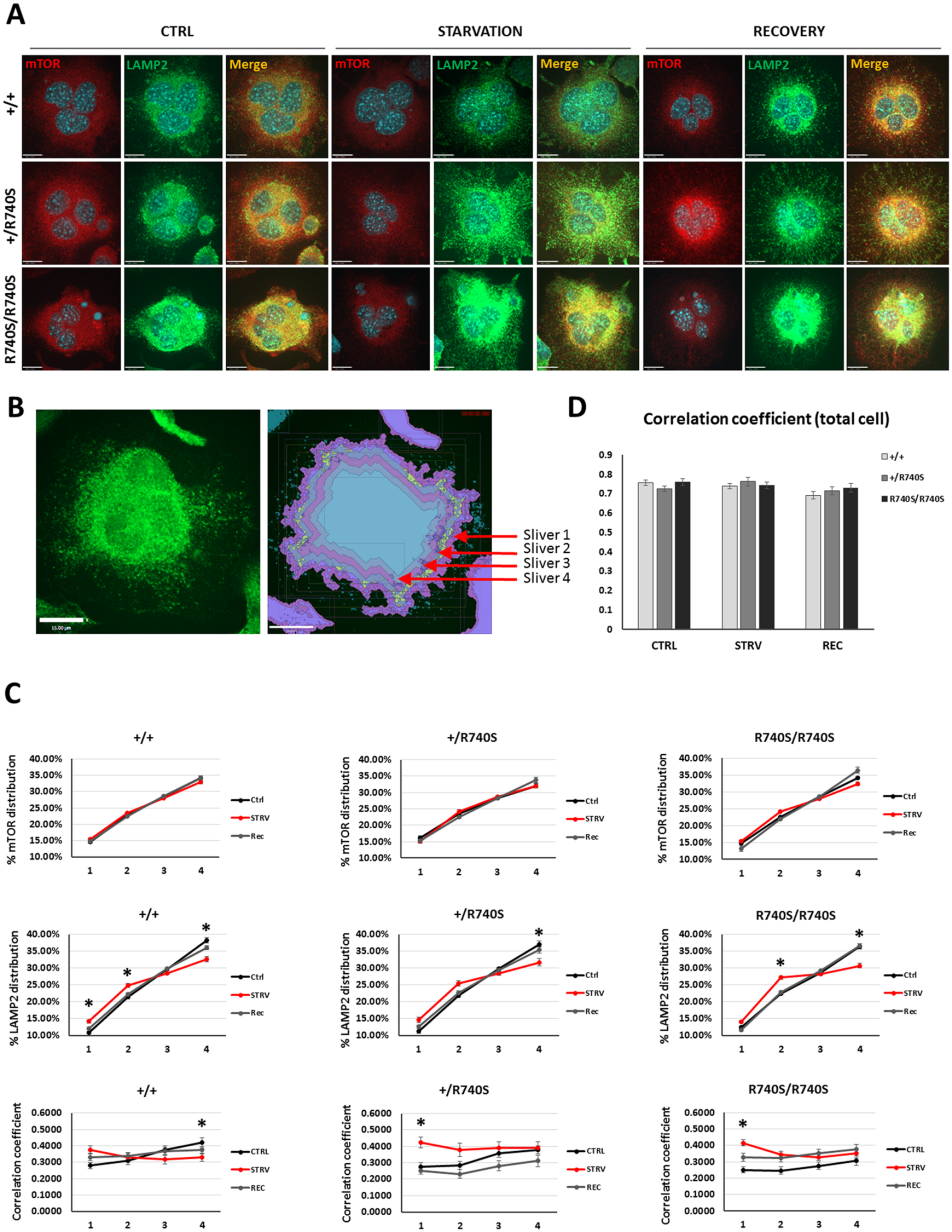
mTOR/LAMP2 co-localization in osteoclasts does not change in response to amino acid availability. Spleen-derived osteoclasts were differentiated as described in “Materials and Methods”. On day 4 of culture, the cells were incubated with HBSS (starvation) for 60 min and then with fully supplemented media for 30 min. (**A**) Cells were fixed and stained using anti-mTOR and anti-LAMP2 antibodies. Representative confocal images; nuclei were stained with DAPI; bars 15 µm. (**B**) Custom macro peripheral depth analysis mask showing depth/sliver distribution. (**C**) Quantification of mTOR and LAMP2 distribution, mTOR/LAMP2 co-localization at the periphery, Pearson’s correlation coefficient; mean ± SEM, * indicates statistical significance compared to +/+ control, p < 0.05. (**D**) Total cell Pearson’s correlation coefficient; mean ± SEM. The number of cells used for quantification is as follows: +/+ CTRL n = 38; +/+ STRV n = 39; +/+ REC n = 47; +/R740S CTRL n = 33; +/R740S STRV n = 27; +/R740S REC n = 34; R740S/R740S CTRL n = 44; R740S/R740S STRV n = 43; R740S/R740S REC n = 45).

**Figure 4:**
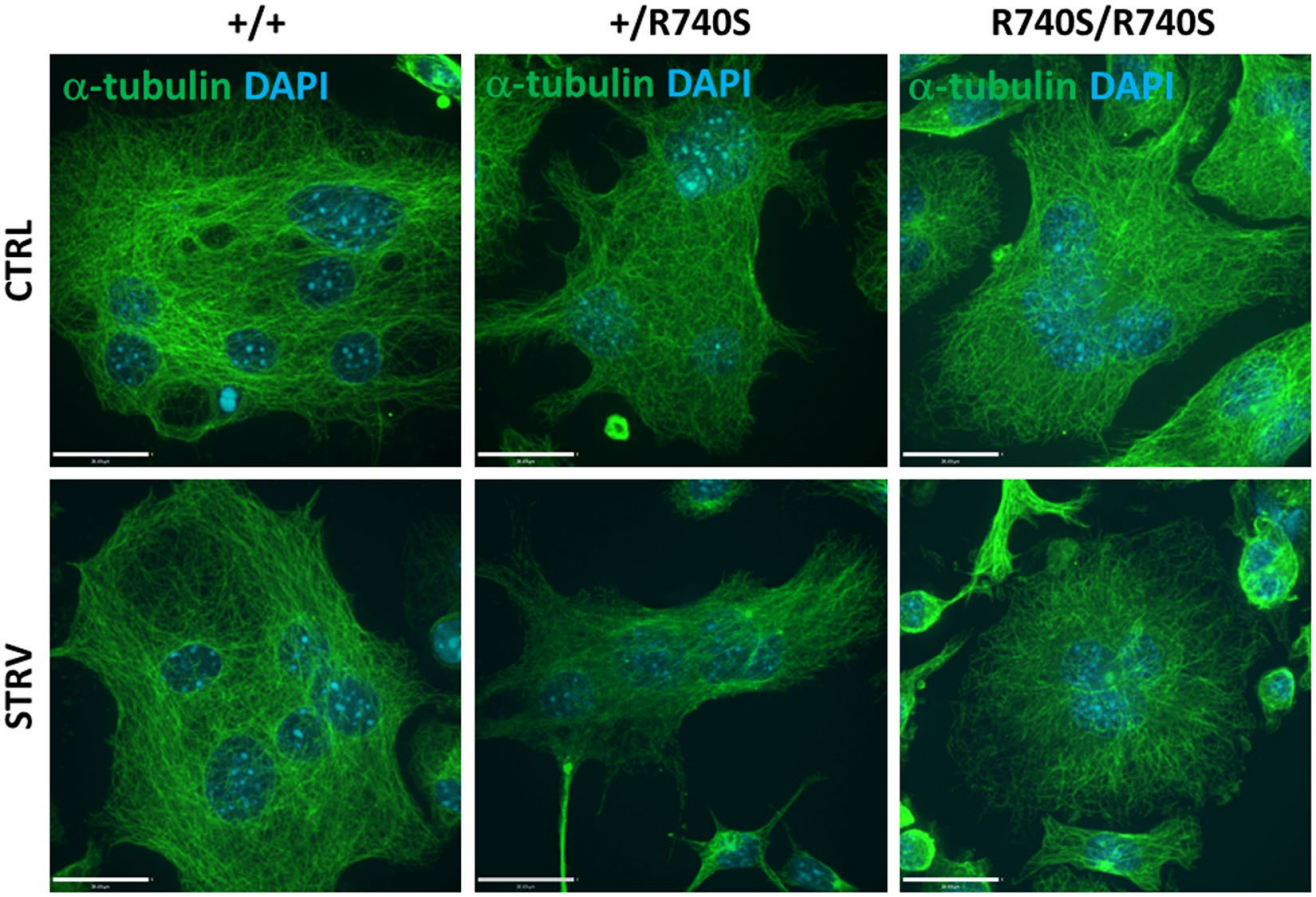
Microtubular organization in osteoclasts. Cells were cultured as described in “Materials and Methods” and stained using anti-α-tubulin antibody; nuclei were stained with DAPI. STRV = starvation, HBSS, 1 hr. Representative images of three independent experiments; bars 20 µm.

It has been shown in HEK-293T cells that active mTORC1 resides on the lysosome and that inactive mTORC1 disassociates from the lysosome in the absence of amino acids^4^. Since osteoclasts with the R740S mutation appear to have aberrant mTORC1 signaling (higher basal p-mTOR and p-p70S6K levels in +/R740S and in R740S/R740S cells, respectively), we decided to determine whether mTOR association with the lysosomes was altered in these cells. Therefore, next, we measured mTOR and lysosomal (LAMP2) positioning, as well as mTOR/LAMP2 co-localization in osteoclasts using confocal microscopy during starvation and recovery (Fig. 3A). We noticed that in osteoclasts, the lysosomes were primarily perinuclear at basal conditions and moved to the periphery during starvation, an observation opposite to the one reported for HeLa cells^5^. To quantify mTOR and lysosomal positioning at the periphery of osteoclasts, we utilized a custom macro previously used to characterize and quantify lysosomal movement and redistribution in HeLa cells^19^. This macro generated four depths or ‘slivers’ (Fig. 3B), allowing us to measure LAMP2, mTOR and LAMP2/mTOR co-localization within those ‘slivers’. To our surprise, there was no difference in mTOR peripheral localization between the genotypes or in response to starvation (Fig. 3C). On the other hand, LAMP2 results confirmed our observation that the lysosomes moved to the periphery during starvation; however, this movement was less apparent in +/R740S and R740S/R740S cells (significantly different in sliver 4, and slivers 2 and 4, respectively). Furthermore, peripheral mTOR/LAMP2 co-localization, as measured by Pearson’s correlation coefficient, was only selectively affected by starvation (Fig. 3C): in +/+ cells, co-localization decreased in response to starvation only in the most inner sliver 4, while in the +/R740S and R740S/R740S osteoclasts, mTOR/LAMP2 co-localization was, in fact, increased at the extreme periphery (sliver 1). However, there was no significant difference in mTOR/LAMP2 co-localization at basal, starvation, or recovery conditions when +/+ osteoclasts were compared to +/R740S and R740S/R740S cells (Figure S2). We also measured the ‘total cell’ mTOR/LAMP2 co-localization using Pearson’s correlation coefficient and found that it was not significantly altered in the osteoclasts in all three genotypes in response to starvation conditions (Fig. 3D). These results showed that there was no apparent significant mTOR/LAMP2 dissociation in response to starvation in all three genotypes, even though mTORC1 was not active during starvation conditions (Fig. 1B,C). The results of our confocal experiments also indicated that there was a minimal lysosomal movement in response to starvation/amino acid withdrawal condition, contradicting observations made in HeLa cells^5^. Since lysosomes move along the microtubules in all cells, we next examined the microtubule network in the osteoclasts. Immunofluorescent staining with anti-α-tubulin antibody suggested that multinucleated osteoclasts lacked the microtubule organization center (MTOC). Furthermore, the microtubules in the osteoclasts were organized in a semi-circular rather than linear fashion (Figs 4 and 3S). These results confirm a previous report characterizing differences in microtubule organization in osteoclasts^20^ and, therefore, providing an explanation to the absence of a linear perinuclear-to-periphery movement of the lysosomes in these unique multinucleated cells.

### mTOR is primarily membrane-associated in osteoclasts

**Figure 5:**
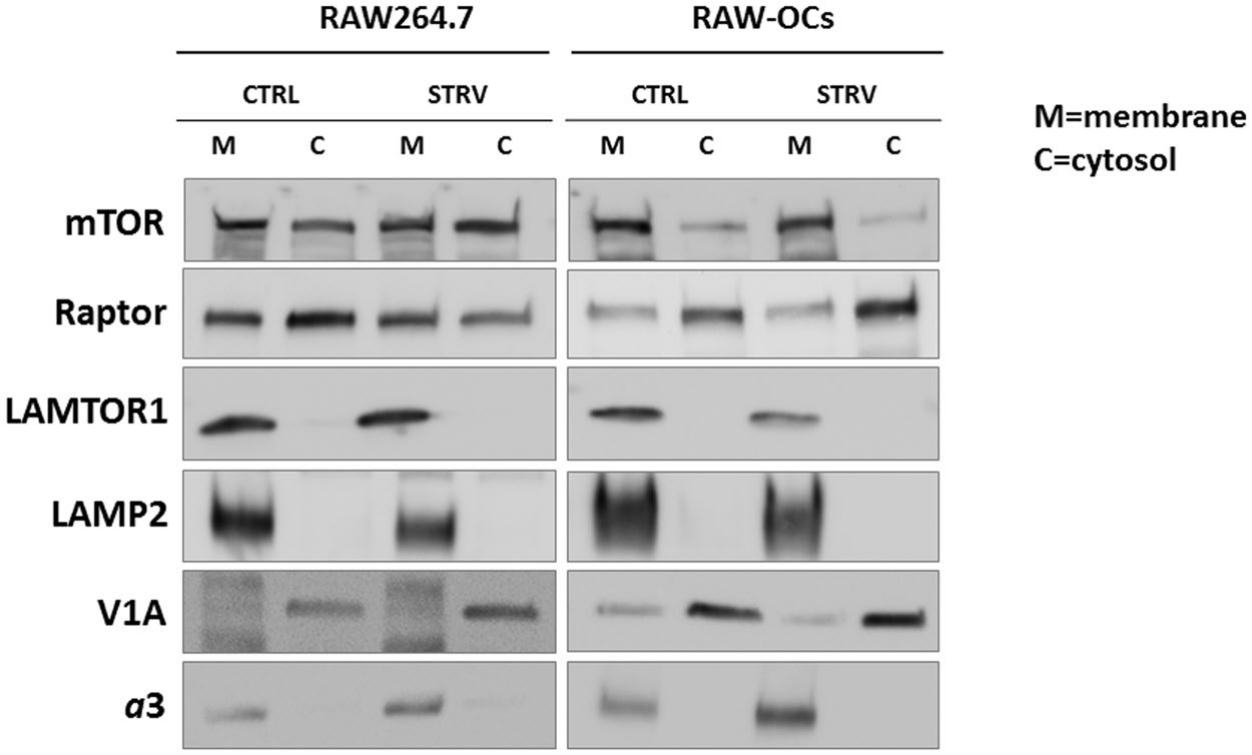
mTOR is primarily membrane-associated in osteoclasts. RAW264.7 cells and RAW-derived osteoclasts (RAW-OCs) were cultured and cytosolic and membrane fractions were prepared as described in “Materials and Methods”. Equal protein amounts were separated on 4–20% gradient gels, transferred to a nitrocellulose membrane and probed for mTOR, Raptor, LAMTOR1, LAMP2, *a*3, and V1A. Representative cropped blots of 4 independent experiments. M = membrane fraction; C = cytosolic fraction; CTRL = control, no treatment; STRV = starvation, HBSS, 1 hr.

Our confocal results showed that there was no difference in a total cell mTOR/LAMP2 co-localization in the presence or absence of amino acids in primary osteoclasts in all three genotypes. This finding appears to contradict a currently accepted model stating that in response to starvation mTOR disassociates from the lysosome and becomes cytoplasmic. To verify our confocal results, we performed fractionation experiments using RAW264.7 cells, a mouse macrophage cell line and a well-established model of osteoclastogenesis. The RAW264.7 cells, when cultured with the receptor activator of NF-κB ligand (RANKL), a cytokine required for osteoclastogenesis, differentiate into fully functional osteoclasts within 5–7 days^21^. Therefore, performing experiments using undifferentiated RAW264.7 (RAW) cells and RAW-derived osteoclasts (RAW-osteoclasts) would allow us to compare mTOR localization in response to starvation in two different cell types. Immunoblots of cytosolic and membrane fractions demonstrated that in undifferentiated RAW cells, mTOR was located both in the cytosolic, as well as in the membrane fraction; however, the starvation appeared to increase cytosolic mTOR levels (Fig. 5). At the same time, in RAW-osteoclasts, the majority of mTOR appeared to be associated with the membrane fraction, while starvation did not affect this membrane/cytosol distribution. We also assessed the localization of Raptor, a key component of mTORC1 complex: Raptor was primarily cytosolic in the RAW-osteoclasts, while it was equally distributed between the cytosol and the membrane fraction in undifferentiated RAW cells. Late endosomal/lysosomal adaptor and MAPK and mTOR activator 1 (LAMTOR1), a Ragulator complex component, was present only in the membrane fraction, confirming previously reported lysosomal association of Ragulator complex with the lysosome^3^. The blots were also probed for the *a*3 and LAMP2 to verify enrichment of the lysosomal fraction. The levels of the V-ATPase V_1_ subunit A (V1A) were also determined, as a representation of the V-ATPase complex assembly. The V1A subunit was primarily cytosolic, as expected, suggestive of V_1_–V_0_ disassociation^22, 23^. Collectively, these fractionation experiments confirmed our confocal results of the absence of mTOR disassociation from the lysosome in response to starvation conditions. These results also suggested that mTOR/membrane association in differentiated multinucleated osteoclasts differs from other cells, such as undifferentiated RAW cells.

### mTOR remains localized to osteoclast lysosomes during starvation

**Figure 6:**
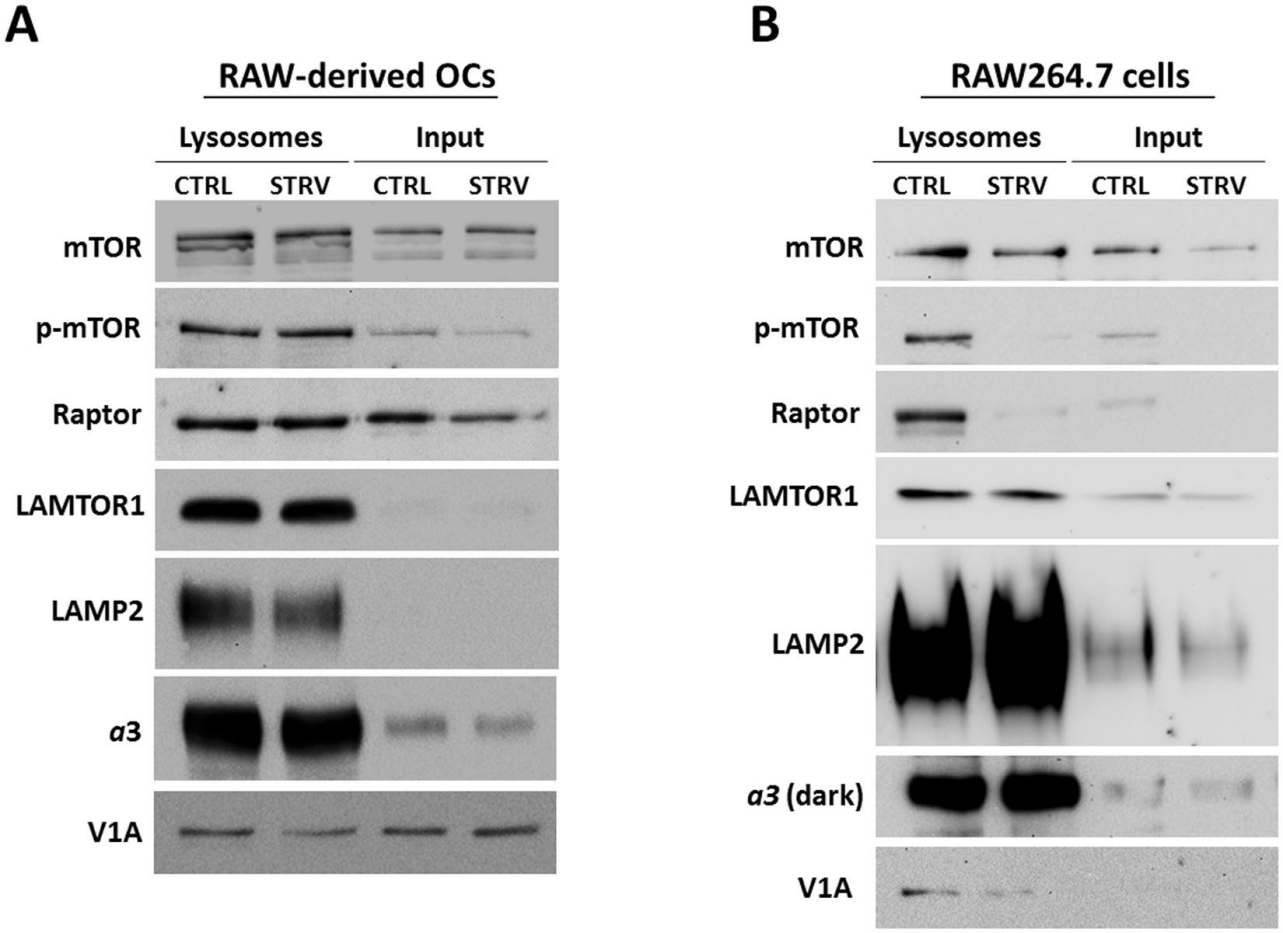
mTOR remains associated with the lysosomes in osteoclasts during amino acid starvation. (**A**) RAW264.7-derived osteoclasts (RAW-OCs) were differentiated for 4 days, incubated with dextran coated magnetic nanobeads for 24 hrs, chased for additional 24 hrs, and lysosomes purified as described in “Materials and Methods”. (**B**) Purified lysosomes from the undifferentiated RAW264.7 cells were isolated as described in “Materials and Methods”. Equal amounts of protein were loaded per lane and the blots were probed for mTOR, p-mTOR, Raptor, LAMTOR1, LAMP2, and the V-ATPase subunits *a*3 and A. Representative cropped blots of three independent experiments for RAW-OCs and for undifferentiated RAW264.7 cells. CTRL = control, no treatment; STRV = starvation, HBSS, 1 hr.

Fractionation experiments demonstrated that mTOR remains membrane-bound in the osteoclasts even during starvation condition; however, the membrane fractions are not pure lysosomes but rather are a collection of all membranes, including plasma membrane. To focus only on the lysosomes, we utilized a well characterized protocol to isolate ultrapure lysosomal fractions using magnetic dextran-coated nanobeads^24^ and assessed mTOR distribution in RAW-derived osteoclasts and in non-differentiated RAW264.7 cells in response to starvation conditions. Similar to the fractionation experiments, RAW-derived osteoclast lysosomes were probed for mTOR and showed that there was no discernable difference in mTOR distribution between control and starvation conditions (Fig. 6A). Phosphorylated (Ser2448) mTOR was also present on osteoclast lysosomes and the p-mTOR levels did not change in response to starvation, even though the total (input) p-mTOR levels did decrease, similar to our previous observations (Fig. 1B,C). In contrast, during starvation, RAW264.7 lysosomes had lower levels of both mTOR and p-mTOR compared to the controls (Fig. 6B). Raptor, a key component of mTORC1 complex, was affected in a similar fashion: starvation decreased lysosome-associated Raptor levels in RAW264.7 cells, but not in RAW-derived osteoclasts, suggesting that the whole mTORC1 complex disassociates in undifferentiated RAW264.7 cells, but remains associated with the lysosome in osteoclasts. LAMTOR1, a Ragulator complex component^3^, was also present in lysosomal fractions, and its levels were not affected by amino acid removal in both cell types. LAMP2 and *a*3 proteins confirmed the enrichment for these proteins in the lysosomal fractions, while presence of V1A indicated an assembled V-ATPase complex (Fig. 6A,B). In summary, these lysosomal fractionation results indicate that in specialized cells such as osteoclasts, the mTOR remains associated with the lysosome even during starvation/amino acid withdrawal conditions.

## Discussion

Due to its prominent role in cell growth, proliferation and differentiation, regulation of mTORC1 is intensively studied. Here, we demonstrate that in osteoclasts, mTOR and mTORC1 complex remain associated with the lysosome even during starvation conditions. Our findings suggest that the mechanisms of mTORC1 regulation and its association with lysosomes are not universal. Our studies point to cell type-specific mechanisms that regulate mTORC1 activity.

Prior studies indicate that at least three factors play a role in mTORC1 activation: (1) starvation or nutrient/amino acid status, (2) the V-ATPase activity, and (3) lysosomal positioning. Therefore, we set out to elucidate the role of the V-ATPases and lysosomal pH in mTORC1 activation and signaling using primary osteoclasts from mice with the V-ATPase *a*3 R740S mutation, utilizing a unique property of these cells – a gene-dosage dependent high lysosomal pH^16^. To confirm that lysosomal function was affected in +/R740S and R740S/R740S osteoclasts, the levels of autophagy markers LC3-II and p62 were assessed (Fig. 2). The apparent absence of autophagic induction in +/+ osteoclasts was likely due to short incubation time point selected. However, 2-hour chloroquine treatment increased LC3-II and p62 levels in +/+ cells, confirming inhibition of the autophagic flux in these cells, but failed to do so in R740S/R740S cells (Figure S1). As expected, starvation abrogated mTORC1 signaling in osteoclasts in all three genotypes; however, the V-ATPase R740S mutation had a differential effect on mTORC1 activation. In +/R740S cells (lysosomal pH ~5.7), p-mTOR basal levels were increased compared to the +/+ controls (lysosomal pH ~4.7), while in the R740S/R740S cells (lysosomal pH ~6.3) the p-mTOR levels were no different from the +/+ control. At the same time, upon recovery, the p-mTOR levels were significantly increased in +/+ cells (compared to basal levels), but not in both +/R740S and R740S/R740S osteoclasts (Fig. 1). These results almost mirrored the upstream AKT activation data: AKT phosphorylation was significantly decreased in +/R740S and R740S/R740S cells upon recovery, compared to both corresponding basal p-AKT levels and to +/+ controls. Collectively, these results suggest that the V-ATPases and the lysosomal pH may play separate roles in initiation (e.g. plasma membrane-associated V-ATPases contribute to AKT phosphorylation) and progression (e.g. lysosomal V-ATPases contribute to mTOR and p70S6K phosphorylation) of mTORC1 signaling pathway.

Lysosomal positioning is one of the factors reported to dictate mTORC1 activity^5^. We observed that at a steady-state, osteoclasts had perinuclear distribution of the lysosomes, opposite to what has been shown in HeLa cells^5^. Endogenous perinuclear lysosomes have been demonstrated in other cells, such as human adipose microvascular endothelial cells and primary human dendritic cells^19^. To assess whether higher lysosomal pH affected lysosomal positioning in osteoclasts, we quantified lysosomal distribution and lysosome/mTOR co-localization using confocal microscopy. To our surprise, there was no difference in lysosomal distribution between all three genotypes at all three experimental (basal, starvation, recovery) conditions (Fig. 3C,D, Figure S2); however, within each genotype, starvation condition did have an effect on lysosomal distribution. We expected to see differences in lysosomal distribution between the genotypes, particularly since it has been reported that the lysosomes have different pH depending on localization within the cell, with more acidic lysosomes around the nucleus and less acidic lysosomes close to the periphery^19^; however, all three genotypes displayed the same pattern of lysosomal distribution.

The major surprise was the finding that mTOR/LAMP2 co-localization in osteoclasts did not change during starvation conditions (Fig. 3D). Fractionation and ultrapure lysosomal purification experiments confirmed that in osteoclasts both mTOR and phosphorylated mTOR remained associated with the lysosomal fraction even during starvation conditions (Figs 5 and 6A), even though mTORC1 signaling was inhibited (Fig. 1B,C). At the same time, mTOR did disassociate from the lysosomes in undifferentiated RAW264.7 cells in response to starvation (Figs 5 and 6B), suggesting that this activation-independent mTOR-lysosome association is specific to the osteoclasts. Based on lysosome-associated Raptor levels, possibly the whole mTORC1 complex either remained attached to the lysosome or disassociated from the lysosome, in osteoclasts and RAW264.7 cells, respectively.

Osteoclasts are highly specialized, terminally differentiated, multinucleated cells performing a unique function in the body – resorption of a mineralized bone matrix. To resorb, osteoclasts polarize on the bone surface and form a ‘ruffled border’ membrane, an organelle similar in properties and contents to the late-endosomal/lysosomal compartment^25, 26^. The resorption itself is a dynamic process that involves continuous and perfectly balanced membrane turnover at the ruffled border: first, the exocytosis of vesicles filled with matrix-degrading enzymes, such as cathepsin K, and, second, simultaneous endocytosis of the degraded material and transport of the bone matrix fragments to the opposite side of the cell (functional secretory domain) *via* transcytosis^9, 27^. Microtubular network plays an important role in this process: it is responsible for transporting vesicles to and from the ruffled border area, and inhibition or disruption of the microtubules leads to inhibition of bone resorption^28^. Furthermore, depending on osteoclast status (resorbing *vs*. non-resorbing), microtubule organization changes. In non-resorbing osteoclasts, or in the cells grown on plastic or glass, the microtubules appear to be randomly organized and do not form typical microtubule asters like seen in mononucleated cells (Figure S3), but, instead, could be juxtanuclear or nucleating in the vicinity of nuclear surfaces and radiating to the cell periphery (Figs 4 and S3). In contrast, in actively resorbing osteoclasts, microtubules are completely reorganized, radiating from the nuclear surfaces towards the ruffled border, and playing a key role in delivery of osteolytic cargo to the resorption site^29^. In this manuscript, we only examined lysosomal positioning and mTOR/lysosome co-localization in non-resorbing cells; however, based on differences in microtubule organization between resorbing and non-resorbing cells, mTOR association with the lysosome might be different during active bone resorption.

Resorption of a highly mineralized matrix, such as bone, is a metabolically demanding process. To perform this unique function and to meet this high metabolic demand, osteoclasts express large number of the V-ATPases necessary for efficient acidification of the resorption lacunae, and a corresponding high number of mitochondria to produce sufficient amounts of ATP to ‘operate’ these pumps (osteoclasts are also called mitochondria-rich cells^30^). It is possible that to address this high metabolic demand (to satisfy mitochondria, the V-ATPase proton translocation, and protein synthesis needs), osteoclasts may require sustained mTOR presence on the lysosome even during starvation; however, the exact mechanism of this osteoclast-specific mTOR-lysosome association is not clear at this point. There are a couple of possibilities: (1) an existence of an osteoclast-specific adaptor molecule that maintains mTOR attachment and/or activates osteoclast-specific signaling pathways; and (2) mTOR lysosomal degradation as a mechanism of inhibition of mTOR activity, since this lysosomal fraction-associated mTOR could be located either outside or inside the lysosome. This second hypothesis is supported by our previous observations that treatment of osteoclasts with the lysosomal inhibitors leads to accumulation of mTOR^17^, suggesting that in osteoclasts mTORC1 signaling is regulated by lysosomal degradation, as well as by a recent study, where the authors observed mTOR lysosomal-mediated degradation in primary hippocampal neurons, also highly specialized cells^31^.

Understanding the mechanisms of mTOR regulation in osteoclasts is very important: mTOR is indispensable for osteoclast differentiation – osteoclastogenesis is impaired in cells deficient for mTOR or Raptor^32^, and treatment of the precursors with mTOR inhibitor rapamycin decreases osteoclast formation^32, 33^. Understanding mTOR signaling in osteoclasts also has significant clinical implications: osteoclasts from patients with rheumatoid arthritis, an autoimmune disease characterized by overactive osteoclasts and pathological bone loss, have increased mTORC1 activity^34^, making mTORC1 signaling pathway a therapeutic target to treat diseases that involve overactive osteoclasts. In fact, everolimus is already used to treat rheumatoid arthritis and other autoimmune diseases^2^; however, the exact mechanism of action of mTOR inhibitors in these diseases still needs to be elucidated.

## Materials and Methods

### Animals

R740S osteopetrotic mice were generated as described previously^14^. Male and female heterozygous mice carrying the R740S mutation (+/R740S) were bred to produce homozygous (R740S/R740S) animals; +/+, +/R740S, and R740S/R740S littermates were used in experiments described below. Animals were genotyped using a custom TaqMan SNP Genotyping protocol (Invitrogen). All experimental procedures received approval by the Toronto Centre for Phenogenomics and the University of Toronto Animal Care Committees, and were conducted in accordance with the guidelines of the Canadian Council on Animal Care.

### Osteoclast cultures

Due to absence of bone marrow and an early lethality of R740S/R740S animals (postpartum days 12–14), osteoclasts were generated using mouse splenocytes as previously described^16^. Briefly, spleens from 5- or 6-days-old pups were dissected, crushed through a sterile cell strainer (100 µm nylon mesh) in α-MEM containing 10% FBS and antibiotics (10 µg/ml penicillin/streptomycin, 10 µg/ml gentamicin, 0.25 µg/ml amphotericin B (Life Technologies)). Cells were centrifuged at 1,000 rpm for 5 min, re-suspended in 200 µl of PBS per spleen, and the red blood cells (RBC) were lysed in freshly made RBC lysis buffer (0.14 M NH_4_Cl, 0.017 M Tris pH 7.65). After 10 min, α-MEM containing 10% FBS and antibiotics was added to stop the lysis, the cells were centrifuged for 5 min at 1,000 rpm, and then washed 3x in PBS using 5 min/1,000 rpm settings. After the final wash, the cell pellet was re-suspended in α-MEM containing 10% FBS and antibiotics, cells were counted using hemocytometer and plated at 1 × 10^6^ cell/ml in α-MEM containing 10% FBS, antibiotics, 25 ng/ml M-CSF (R&D Systems) and 100 ng/ml RANKL (made in-house). For immunoblotting, cells were plated in 12-well plates at 1 ml/well; for immunofluorescence/confocal experiments, the cells were plated in 8-well chamber slides (BD Falcon) at 0.4 ml/well. Cell were cultured for 4 days at 37 °C in 5% CO_2_ with media change on day 2.

### Starvation and recovery experiments

Spleen-derived osteoclasts were cultured as described above. For starvation experiments, on the last day of the culture the cells were washed 2 times with warm HBSS (Gibco/Life Technologies) to remove traces of amino acids and serum, and then incubated at 37 °C in 5% CO_2_ for 1 hr. For recovery experiments, the cells were washed 2 times with warm HBSS, incubated with HBSS for 1 hr at 37 °C in 5% CO_2_ and then treated with fully supplemented media containing 12.5 ng/ml M-CSF and 50 ng/ml RANKL for 30 min. The cells were then either fixed for immunofluorescence experiments or lysed for protein analysis (see below).

### Immunoblotting

Spleen-derived osteoclasts were cultured in 12-well tissue culture plates as described above. The plates were washed two times with cold PBS and lysed using RIPA buffer (0.1% triton X-100, 50 mM Tris, 300 mM NaCl, 5 mM EDTA), containing protease inhibitors (protease inhibitors cocktail P8340 (Sigma Aldrich) and 1 mM PMSF), and phosphatase inhibitors (Sigma Aldrich, P5726). Whole cell lysates were separated on 4–20% gradient TGX gels (BioRad), transferred to nitrocellulose membrane and probed for LC3B, p62, *a*3, LAMP2, mTOR, p-mTOR (S2448), p-Akt (S473), pan-AKT, p-p70S6K (T389) and actin (all from Cell Signaling Technologies (CST), except *a*3 (custom made^15^) and LAMP2 (Developmental Studies Hybridoma Bank, Iowa, #ABL-93c)). Amersham ECL Prime Western blotting reagent was used as a detection reagent. Images were captured using BioRad ChemiDoc Gel Docking system.

### Confocal microscopy

Spleen-derived osteoclasts were cultured as described above. For LC3 staining (CST, #2775), cells were fixed for 30 min at 37 °C in 4% paraformaldehyde (PFA) in 80 mM Hepes (pH 7.2) with 6.8% sucrose and permeabilized using cold methanol for 10 min as per manufacturer’s instructions. For mTOR (CST, #2983) and LAMP2 (Developmental Studies Hybridoma Bank, Iowa, #ABL-93c) staining, cells were fixed for 30 min at 37 °C in 4% PFA in 80 mM Hepes (pH 7.2) with 6.8% sucrose, permeabilized/blocked for 1 hr in 0.3% Triton-X, 5% normal goat serum (NGS) and 1% BSA in PBS. The samples were then incubated with the primary antibodies, anti-mTOR (1:200), anti-LC3 (1:400), and anti-LAMP2 (1:400), in 1% BSA, 1% NGS and 0.3% Triton-X in PBS for 16 hours, and then with secondary anti-rabbit Alexa Fluor 555 (CST, #4413) or anti-rat Alexa Fluor 488 (CST, #4416) antibody for 1 hr. For microtubules staining, cells were fixed and permeabilized in 4% PFA as described for mTOR/LAMP2 staining, and incubated with anti-α-tubulin antibody (Sigma-Aldrich, T9026; 1:500) overnight, and then with anti-mouse Alexa Fluor 488 secondary antibody (CST, #4408) for 1 hr. The slides were mounted in DAPI (4′6-diamidino-2-phenylindole)-containing mounting media (ProLongGold antifade reagent, Life Technologies) and analyzed using a 63 × 1.35 NA oil-immersion lens using a spinning-disk confocal microscope (Olympus IX81) equipped with an EM-CCD camera (C9100–13, Hamamatsu, Quorum Technologies Inc.) under control of Volocity 6.3.1 software (Perkin-Elmer).

### Membrane/cytosolic fractionation

RAW264.7 cells (ATCC) were cultured in 10% FBS in DMEM (Gibco/Life Technologies) containing 10 µg/ml penicillin/streptomycin. For RAW-derived osteoclasts, RAW cells were cultured in a presence of 200 ng/ml RANKL for 5 days. For fractionation experiments, RAW-derived osteoclasts or RAW cells were cultured in 100 mm dishes, washed with 5 ml of cold PBS twice, and then scraped in 380 µl of homogenization Buffer (205 mM sucrose, 1 mM EDTA and 10 mM HEPES, pH 7.4) per dish (4 × 100 mm dishes/group). For each experimental group, combined cell suspension was homogenized using Dounce homogenizer (tight) with 60 strokes. The homogenate was cleared (from the nuclei and intact cells) by centrifugation at 4 °C, 500 × g for 10 min. After clearing, the homogenate was centrifuged at 100,000 × g for 30 min at 4 °C. Supernatant was removed and represented a cytosolic fraction, while the pellet was re-suspended in homogenization buffer and represented a membrane fraction. SDS was added to the both fractions to a final concentration of 1%. Protein concentration was measured using Bradford-based method (BioRad protein assay reagent, #500–006). Samples (10 µg) from each fraction separated on 4–20% SDS PAGE and transferred to the nitrocellulose membrane. Immunoblotting was performed as described above using mTOR, ATP6V1A (GeneTex), *a*3, LAMP2, Raptor (CST, #2280), and LAMTOR1 (CST, #8975) antibodies.

### Lysosomal purification experiments

Ultrapure lysosomal fractions were prepared using commercially available magnetic nanoparticles (40 kDa EndoMAG-40, Liquids Research Ltd., UK) and the protocol described by Walker *et al*.^24^. Undifferentiated RAW264.7 cells and RAW264.7-derived osteoclasts, an established *in vitro* model of osteoclastogenesis, were used in these experiments. For RAW-derived osteoclast experiments, RAW264.7 cells were cultured in 100 mm dishes at 5 × 10^5^ cells/dish in DMEM (Gibco/Life Technologies) containing 10% FBS, 10 µg/ml penicillin/streptomycin, and 200 ng/ml RANKL for 5 days (10 × 100 mm dishes/group were used in these experiments). On day 5, the cells were incubated with the media containing 10% (v/v) EndoMAG nanoparticles for 24 hr, and then chased for additional 24 hrs. For undifferentiated RAW264.7 cell experiments, 1.5 × 10^6^ cells/100 mm dish in 10% FBS in DMEM and 10 µg/ml penicillin/ streptomycin were plated; 8 × 100 mm dishes/group were used in the experiments. RAW264.7 cells were incubated with 10% (v/v) EndoMAG nanoparticles for 24 hr, and then chased for additional 24 hrs. For starvation experiments, cells were washed 2xHBSS and then incubated in HBSS for 1 hr at 37 °C, 5% CO_2_. Control groups had media replaced with fresh media for the same time period (1 hr). Cells were then harvested by scraping into 4 ml of cold buffer A (15 mM KCl, 1.5 mM MgAc, 1 mM DDT, 10 mM HEPES, 1x proteinase inhibitors cocktail (Sigma-Aldrich)), homogenized with tight Dounce tissue grinder (30 strokes), and passed through a 23 G needle 8 times. Next, 1 ml buffer B (220 mM HEPES pH 7.2, 0.1 mM sucrose, 375 mM KCl, 22.5 mM MgAc, 1 mM DTT, 100 µg/ml DNase I (Sigma-Aldrich)) was added, the homogenate was incubated on ice for 5 min, and then centrifuged for 10 min at 200× g (at +4 °C). The supernatants were loaded on 0.5% BSA equilibrated LS columns (Miltenyi Biotec) mounted on the QuadroMACs magnetic separator (Miltenyi Biotec). The columns were first washed with 1 ml of DNase I solution (100 µg/ml DNase I (Sigma-Aldrich) in 0.1 mM sucrose in PBS), let stand for 10 min at room temperature, and then washed with 1 ml of 0.1 mM sucrose in PBS. The columns were then removed from the magnetic stand and the purified lysosomes were eluted using 500 µL of 0.1 mM sucrose and proteinase inhibitor cocktail (Sigma-Aldrich) in PBS. Multiple elution were used to increase the yield. Protein concentration was determined using Bradford Assay (BioRad). Samples (10 µg/lane for osteoclasts and 5 µg/lane for RAW264.7 cells) were separated on 4–20% gradient gel, transferred to a nitrocellulose membrane, and probed for LAMP2, mTOR, p-mTOR, Raptor, LAMTOR1, and the V-ATPase subunits V1A and *a*3.

### Statistical analysis of lysosomal positioning experiments

For statistical analysis, the confocal images of each of the three separate data sets of mTOR and LAMP2 fluorescence, as well as Pearson’s correlation coefficient as a measurement of mTOR/LAMP2 co-localization were used. Linear regression adjusting for repeated measures (mixed model) was used to model the difference between experimental conditions; p-values were adjusted using the Bonferroni method for multiple comparisons. These statistical analyses were performed using the SAS version 9.4 (SAS Institute, Cary, North Carolina, USA).

### Statistical analysis of combined data of immunoblotting experiments

To address the issue of technical noise when combining the results of independent immunoblotting experiments (relative density of the bands), signal distribution normalization was performed. The intensity of each band for each protein in each group was normalized to the median intensity of its group using the following formula: Normalized Intensity for protein band = (Raw Intensity of protein band/ Total Intensity of all protein bands on the gel) × Median Intensity of all protein bands.

For statistical analysis of the combined data of all immunoblotting experiments SAS General Linear Model (GLM) procedure was used to carry out an analysis of variance (two-way ANOVA 3 × 3 on a completely randomized block) to estimate the effect of genotype and factors such as protein type and time and their interactions. An analysis of residuals led to the adoption of the natural logarithm of the response which reduced their positive skewness and improved the model. When several comparisons were made, the p-values were adjusted using Tukey’s method. Sample size is as indicated in the methods and figures; p values were considered significant at p < 0.05. All analyses were performed using SAS software, V.9.4 (SAS Institute, Cary, North Carolina, USA).

## Electronic supplementary material

Supplemental Info
