# Supplementary material for: Activity-independent targeting of mTOR to lysosomes in primary osteoclasts
Source: Sci Rep. 2017 Jun 7;7:3005. doi: 10.1038/s41598-017-03494-2 (PMC5462732; doi:10.1038/s41598-017-03494-2)
Supplement: Supplementary file 1 — Supplemental Info [file 41598_2017_3494_MOESM1_ESM.pdf]

Supplemental Info

Manuscript title: "Activity-independent targeting of mTOR to lysosomes in primary osteoclasts"

Authors: Andrew Wang, Luciene R. Carraro-Lacroix, Celeste Owen, Bowen Gao, Paul N. Corey, Pascal Tyrrell, John H. Brumell, Irina Voronov.

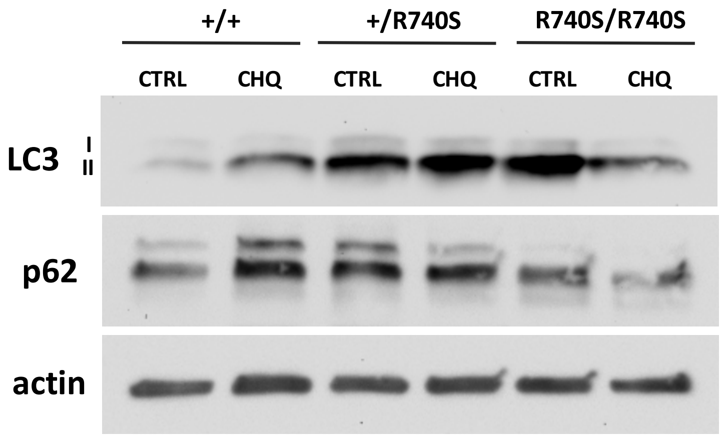

Figure S1. Spleen-derived osteoclasts were differentiated as described in "Materials and Methods". On day 4 of culture, the cells were incubated with 20 μM chloroquine (CHQ) for 2 hrs. Whole cell lysates were separated on 4-20% gradient gels, transferred to a nitrocellulose membrane and probed for LC3, p62 and actin. Immunoblotting, representative cropped blots of 2 independent experiments.

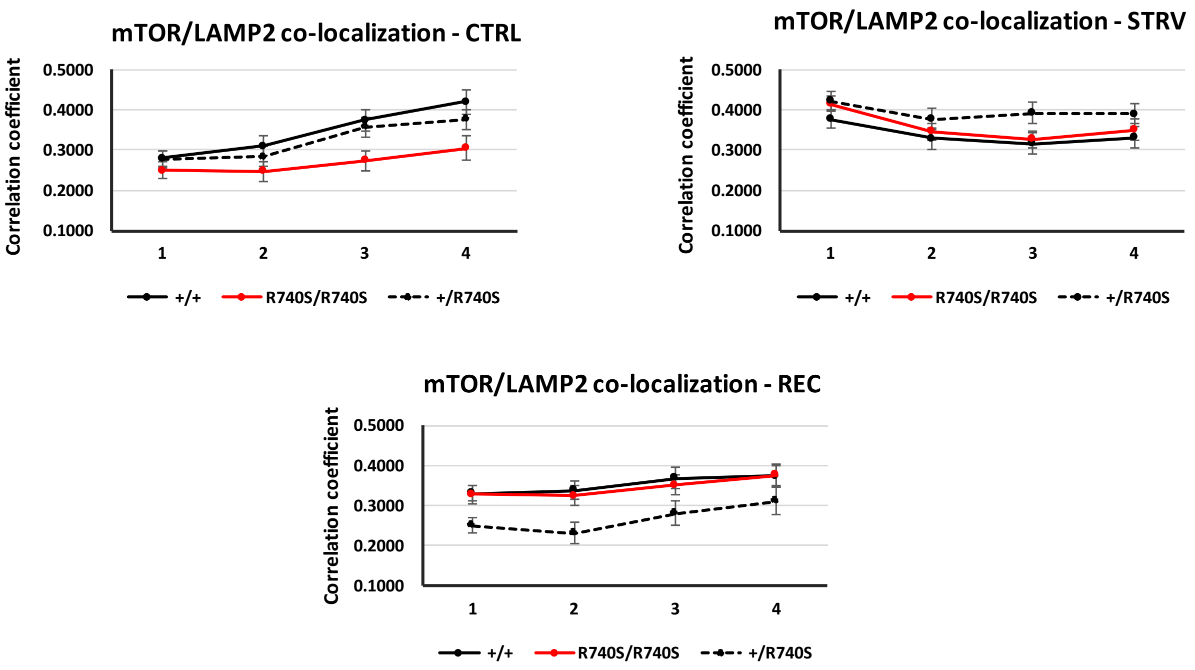

Figure S2. Spleen-derived osteoclasts were differentiated as described in "Materials and Methods". On day 4 of culture, the cells were incubated with HBSS (starvation) for 60 min and then with fully supplemented media for 30 min. Cells were fixed and stained using anti-mTOR and anti-LAMP2 antibodies. Quantification of mTOR and LAMP2 Pearson's correlation coefficient using Volocity custom macro peripheral depth analysis mask showing depth/sliver distribution as described in detail in "Materials and Methods" section. CTRL=control condition, no treatment; STRV=starvation; REC=recovery. The results are plotted as mean ± SEM. The number of cells used for quantification is as follows: +/+ CTRL n=38; +/+ STRV n=39; +/+ REC n=47; +/R740S CTRL n=33; +/R740S STRV n=27; +/R740S REC n=34; R740S/R740S CTRL n=44; R740S/R740S STRV n=43; R740S/R740S REC n=45). There was no significant difference between all three genotypes neither at a steady-state condition, nor in response to starvation and recovery (+/+ CTRL S4 vs. R740S/R740S CTRL S4 p= 0.0696).

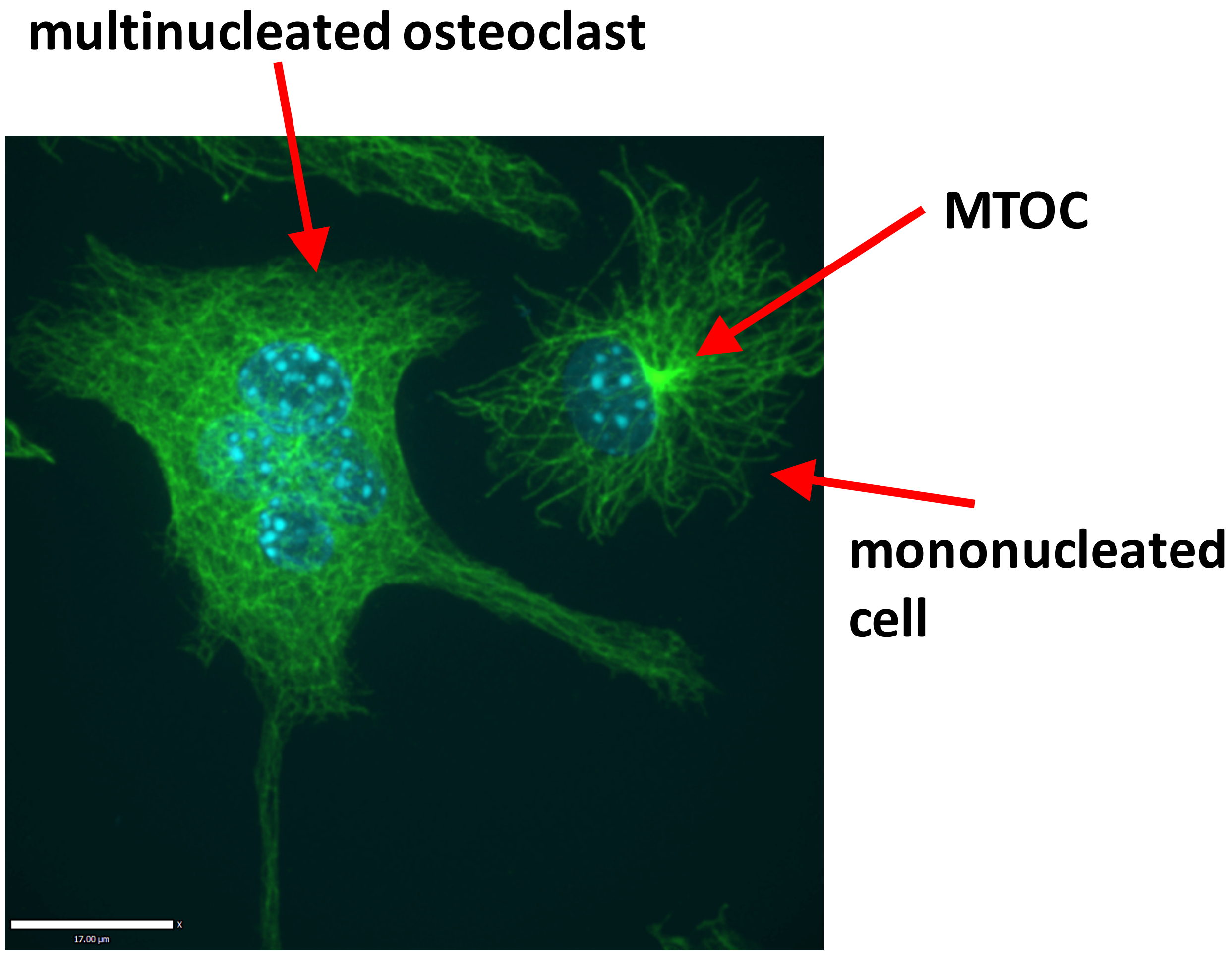

Figure S3. Cells were cultured as described in "Materials and Methods" and stained using anti-α-tubulin antibody; nuclei were stained with DAPI. Representative image of control (steady state) mononucleated and multinucleated cells from +/R740S spleen cultures. These cells show microtubular organization typical of mononucleated cells (microtubule aster, with microtubules radiating from MTOC) and multinucleated osteoclasts (microtubules are juxtanuclear and have concentric appearance). Bars 17 µm.
